# Enabling Informed Decision Making in the Absence of Detailed Nutrition Labels: A Model to Estimate the Added Sugar Content of Foods

**DOI:** 10.3390/nu15040803

**Published:** 2023-02-04

**Authors:** Reka Daniel-Weiner, Michelle I. Cardel, Michael Skarlinski, Angela Goscilo, Carl Anderson, Gary D. Foster

**Affiliations:** 1 WW International, Inc., New York, NY 10100, USA; 2 Department of Health Outcomes and Biomedical Informatics, College of Medicine, University of Florida, Gainesville, FL 32610, USA; 3 Center for Integrative Cardiovascular and Metabolic Disease, University of Florida, Gainesville, FL 32611, USA; 4 Center for Weight and Eating Disorders, Perelman School of Medicine, University of Pennsylvania, Philadelphia, PA 19104, USA

**Keywords:** added sugar, diabetes, obesity, informed decisions, machine learning in nutrition

## Abstract

Obesity and diabetes have emerged as an increasing threat to public health, and the consumption of added sugar can contribute to their development. Though nutritional content information can positively influence consumption behavior, added sugar is not currently required to be disclosed in all countries. However, a growing proportion of the world’s population has access to mobile devices, which allow for the development of digital solutions to support health-related decisions and behaviors. To test whether advances in computational science can be leveraged to develop an accurate and scalable model to estimate the added sugar content of foods based on their nutrient profile, we collected comprehensive nutritional information, including information on added sugar content, for 69,769 foods. Eighty percent of this data was used to train a gradient boosted tree model to estimate added sugar content, while 20% of it was held out to assess the predictive accuracy of the model. The performance of the resulting model showed 93.25% explained variance per default portion size (84.32% per 100 kcal). The mean absolute error of the estimate was 0.84 g per default portion size (0.81 g per 100 kcal). This model can therefore be used to deliver accurate estimates of added sugar through digital devices in countries where the information is not disclosed on packaged foods, thus enabling consumers to be aware of the added sugar content of a wide variety of foods.

## 1. Introduction

Ultra-processed food, and more specifically, the added sugar within, is considered to be a key contributor toward poor nutrition and diet that can lead to the development of both obesity and diabetes [1,2,3]. The 2020–2025 Dietary Guidelines for Americans recommend that added sugar intake be limited to 10 percent of the daily calories consumed, yet the average added sugar consumption per day is greater than 13 percent and most commonly comes from sugar-sweetened beverages [4]. Additional common sources of added sugar include desserts and sweet snacks, coffee and tea, candy, breakfast cereals and bars, sandwiches, and higher fat milk and yogurt [4]. Between 2000 and 2013, the number of available products with added sugar, particularly beverages, significantly increased [5]. In response, the U.S. Food and Drug Administration amended the Nutrition Facts label in 2016 to include information about added sugars and required that all labels present this information to consumers by 1 January 2021 [6]. This represented the first update to the Nutrition Facts label in over 20 years [4].

Many countries across the world have started labeling sugar information on packaged products; however, to date only a few countries include added sugar in addition to total sugar on the Nutrition Facts label [7]. The lack of access to information about added sugars in most parts of the world can create difficulties among consumers attempting to make an informed decision regarding which products to purchase and/or consume. On the other hand, a growing proportion of the world’s population has access to mobile devices, which opens the potential for digital solutions that support health decisions [8].

Previously published approaches to estimate the added sugar content of foods are often time-intense and rely on several, often manual, steps that provide challenges when incorporating them into an automated process [9,10,11]. More recently, fully automated supervised machine learning-based approaches have been successfully employed to estimate nutrient profiles [12,13,14,15]. Most importantly, Davies et al. [15] showed that an approach using the k nearest neighbors (kNN) method can be successfully used on a curated dataset which includes category labels and complete ingredient information. However, the kNN algorithm, which automatically determines the value of the variable to predict from its closest neighbors in the feature space, is known to be susceptible to outliers in the dataset and does not scale particularly well due to the need to store all training examples and find the nearest neighbors at prediction time [16].

A more recent algorithm based on gradient boosted trees, the XGboost algorithm, has been widely recognized for its superior performance in supervised machine learning problems. For example, in the 2015 KDD cup, an annual data modeling competition, all top 10 entries used XGboost in their models [17]. XGboost is a tree-based algorithm, meaning that classification and regression trees (CART) are built out to predict the value of the dependent variable. For example, one branch of a tree could state that for a 100 g portion of a food, if the value of sugar is larger than 15 g, and the value of protein is smaller than 10 g, we expect the added sugar value to be 8 g on average. In the gradient boosting step, additional trees are built to minimize the errors from previous trees, resulting in a model that combines the outcome of many (typically hundreds or thousands) of single trees. The XGBoost algorithm has several optimizations that make it less prone to overfitting the training data and improve its scalability [17].

The objective of the current study was to train an XGBoost model to predict the added sugar content of foods in a production environment where the estimation of added sugar relies directly on input from users to make predictions (i.e., when extensive data cleaning and validation are not possible, complete ingredient labels cannot be input, reliable category information is not available, and prediction algorithms have to be scalable), and examine whether the achievable prediction accuracy allows for making clinically relevant predictions. We hypothesized that food containing high amounts of added sugar would have differing macro- and micronutrient profiles, relative to whole and/or unprocessed foods. Therefore, we expected that added sugar values could be estimated from other nutritional information contained in the nutrition label, such as sugar and protein [15]. Additionally, we expected the relationship between added sugar and other nutrients to be nonlinear and to contain interactions; therefore, we chose an XGBoost algorithm to predict added sugar values, which handles these conditions well [17], and compared the results to the outcome of a previously employed kNN algorithm [15].

## 2. Materials and Methods

We collected nutrition data with complete nutrition label information, including added sugar values, for 69,769 foods from the U.S. portion of the WW International Inc. food database to train models to estimate their added sugar content. The mean values for this overall dataset per default serving size were 1.31 g of added sugar, 0.13 g of alcohol, 9.7 g of carbohydrates, 0.93 g of fiber, 4.74 g of protein, 2.11 g of sugar, 206.69 mg of sodium, 4.59 g of total fat, and 1.45 g of saturated fat, and an average of 99.05 kcal.

To evaluate the accuracy of the resulting models, we generated an unbiased test sample by stratifying the available data on consumption frequency, and randomly sampled 20% of the data to be held out of the model training process. The dataset also included category labels, which were only used to perform an in-depth analysis of error rates on the main sources of added sugar as identified by the USDA, as this category information was not expected to be reliably present at prediction time (when users of the algorithm were inputting only information directly available on the packaging). Thus, 80% of the data were used to train and optimize the model. We used calories, grams of carbohydrates (without fiber or sugar alcohols), grams of total fat, grams of saturated fat, grams of sugar, grams of fiber, grams of protein, and milligrams of sodium in a single default portion size as features to predict the value of added sugar in each default portion size. This information, along with the added sugar content, is available on U.S. packaged foods (https://www.ecfr.gov/current/title-21/chapter-I/subchapter-B/part-101/subpart-A/section-101.9, accessed on 27 January 2023), while only the predictors might be available in other countries, such as those within the EU (https://eur-lex.europa.eu/legal-content/EN/TXT/?uri=CELEX%3A02011R1169-20180101, accessed on 27 January 2023).

For the kNN algorithm, we used 4-fold cross-validation within the training dataset, and a grid search over the hyperparameters as reported by Davies et al. [15], i.e., over the number k of nearest neighbors considered (1,3,5,8, or 10), the distance metric (Euclidean or Manhattan distance), and the weighting of neighbors (uniform or distance-based weighting). Similarly, as for the XGBoost algorithm, and in contrast to the methods reported by Davies et al. [15], we only included nutrient information and no category or ingredient information, as we did not expect these to be available at prediction time in an environment where the user had to input all necessary information for the prediction.

For the XGBoost algorithm, we also used 4-fold cross-validation within the training dataset, and performed a grid search over the hyperparameter space for maximum tree depth (10, 20, or 30), learning rate (0.005, 0.1, or 0.01), minimum child weight (3, 5, or 8), the subsample ratio of columns when constructing each tree (0.5, 0.75, or 0.9), the subsample ratio of the training data (0.25, 0.5 or 0.75), and the number of trees (1000, 2000, or 3000) to determine the optimal set of hyperparameters (detailed documentation on parameters for XGBoost is available at https://xgboost.readthedocs.io/en/stable/parameter.html, accessed on 27 January 2023).

While predictions of the kNN algorithm can easily be derived from averaging values of similar foods, the XGBoost algorithm relies on combining the output of thousands of individual trees, making the reasons for a specific prediction harder to visualize. To nevertheless achieve explainability and help develop an intuitive understanding of the nutrient relationships detected by the final model, we used SHAP (SHapley Additive exPlanations) values, a game-theoretic approach to evaluate the importance of each feature and its relationship to other features in the XGBoost model [18].

The remaining 20% of the data were used as the test dataset to report all accuracy results. In order to facilitate comparisons with different datasets where default portion sizes might be recorded differently, all data in the test dataset were scaled to 100 kcal for normalized reporting of results, and we excluded test data for foods where the default portion size was less than 1 calorie to avoid errors due to scaling. The results were reported both for 100 kcal servings as well as for the default serving size. While we included all foods in the training data to allow the model to learn potential interactions between all nutrients, all foods with zero total sugar were excluded from the test dataset, as correctly predicting added sugars in these foods is trivial. This cleaning of the test dataset resulted in a test dataset size of 4295 observations. Category labels in our dataset that overlap with main sources of added sugar as identified by the USDA included “Beverages” (4.5% of test data), “Desserts, cakes, candy, cookies” (8.8% of test data), ”Breakfast cereal, pancakes and waffles” 3.5% of test data), and “Dairy” (8.5% of test data). The mean value of added sugar was M = 7.62 g (SD = 13.59) per default portion size and M = 6.68 g (SD = 10.04) per 100 kcal for “Beverages”; M = 10.39 g (SD = 6.44) per default portion size and M = 7.44 g (SD = 4.68) per 100 kcal for “Desserts, cakes, candy, cookies”; M = 8.08 g (SD = 6.18) per default portion size and M = 4.64 g (SD = 2.93) per 100 kcal for ”Breakfast cereal, pancakes and waffles”; and M = 2.76 g (SD = 3.91) per default portion size and M = 3.85 g (SD = 5.22) per 100 kcal for “Dairy”. Mean added sugar in the overall test dataset was 4.26 g (SD = 8.14) per default portion size and 3.55 g per 100 kcal (SD = 5.39).

Other main categories in the test dataset were “Meat and Poultry” (17.6%), “Snacks” (11.7%), “Vegetables” (9.3%), “Prepared foods, salads and sides” (8.2%), “Bread and baked goods” (5.7%), “Jams, spreads, salsa and dips” (4.4%), “Fruits” (3.3%), “Ingredients” (2.6%), “Condiments, sauces and gravies” (2.4%), “Oils and dressings” (2.3%), “Chain restaurants” (2.0%), “Soups, stews and chili” (1.5%), “Dairy substitutes and meat substitutes (1.5%), “Pasta, Rice and Grains” (1.0%), “Supplements” (0.7%), and “Fish and Seafood” (0.6%). Note that this distribution is skewed away from normal consumption patterns as no foods with zero sugar were included. Including foods with zero sugar would lead to an overestimation of the accuracy of the presented models, as for all foods with zero sugar added, the sugar content also has to be zero, and no additional information can be gained from applying an estimation model. Mean added sugar in the test dataset was 4.26 g (SD = 8.14) per default portion size and 3.55 g per 100 kcal (SD = 5.39).

To evaluate the accuracy of both algorithms, the models obtained after hyperparameter optimization were used to predict added sugar values on the complete test dataset, irrespective of category, and all predictions were truncated to lie between 0 and the value of sugar. The explained variance and mean absolute error were calculated, as well as a rank correlation (Spearman’s rho), to compare the actual to predicted added sugar values.

## 3. Results

### 3.1. Accuracy of Predicting Added Sugar Using the kNN Algorithm

The results of our grid search indicated that on the current training data evaluating 5 nearest neighbors with a Manhattan distance and weighting them based on distance performed best. Using these hyperparameter settings, we trained a kNN model on the training data and predicted added sugar values on the test data.

The evaluation of this resulting model showed that 85.5% of the variance per default portion size was explained (73.4% per 100 kcal). The mean value of estimated added sugar was 3.79 g (SD = 7.64) per default portion size and 3.1 g (SD = 4.89) per 100 kcal, with a mean absolute error of 1.31 g per default portion size and 1.19 g per 100 kcal. For category labels in our dataset that overlapped with the main sources of added sugar as identified by the USDA, the mean value of estimated added sugar was M = 7.91 g (SD =13.09) per default portion size and M = 6.87 g (SD = 9.17) per 100 kcal for “Beverages”; M = 9.88 g (SD = 5.97) per default portion size and M = 6.88 g (SD = 4.07) per 100 kcal for “Desserts, cakes, candy, cookies”; M = 7.12 g (SD = 5.62) per default portion size and M = 4.06 g (SD = 2.74) per 100 kcal for ”Breakfast cereal, pancakes and waffles”; and M = 2.78 g (SD = 3.78) per default portion size and M = 3.69 g (SD = 4.80) per 100 kcal for “Dairy”. Mean added sugar in the overall test dataset was 4.26 g (SD = 8.14) per default portion size and 3.55 g per 100 kcal (SD = 5.39). The rank correlation (Spearman’s rho) between actual and predicted added sugar values was 0.76 (*p* < 0.001) both per default portion size and per 100 kcal.

**Figure 1:**
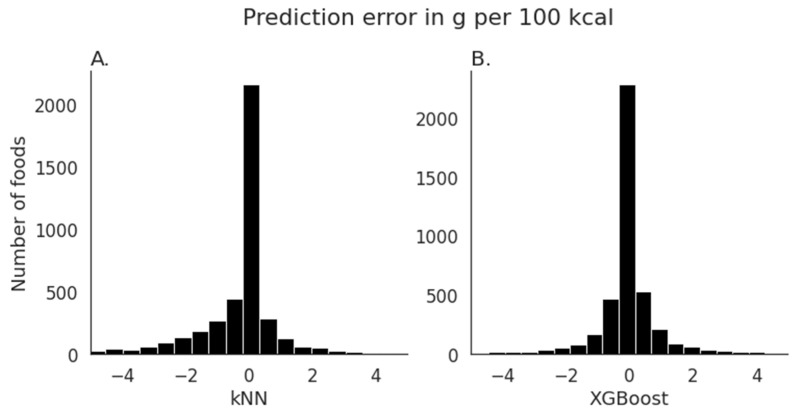
Error in the prediction of added sugar values in g per 100 kcal. (**A**) When using the kNN algorithm for estimation, errors skew towards more errors being < 0 g, indicating that the model tends to underestimate added sugar content. The mean absolute error is 1.19 g. (**B**) When using the XGBoost algorithm for estimation, errors are roughly symmetrically distributed around 0 with a slight bias towards overestimating added sugar content. The mean absolute error is 0.81 g.

**Figure 2:**
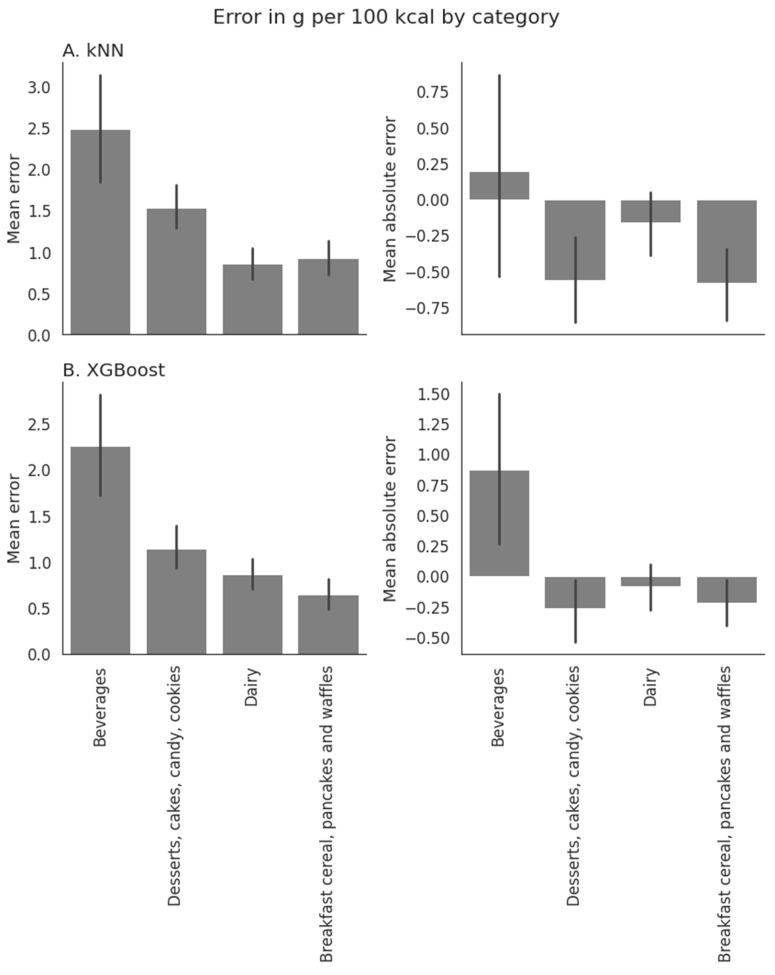
Mean absolute and mean error in the prediction of added sugar values in g per 100 kcal in categories that are high in sugar content for (**A**) the kNN and (**B**) the XGBoost algorithms. The mean error for beverages is positive for both algorithms, while all other categories of interest have negative mean absolute errors, indicating that the added sugar content of beverages is overestimated on average, while added sugar values in the other categories are underestimated on average. Overestimation of added sugar values in beverages can occur, e.g., in fruit juices which are high in natural sugar but low in added sugar.

Figure 1A shows that the distribution of errors in grams per 100 kcal skewed towards more errors < 0 g, indicating that most errors were towards underestimated added sugar values. Figure 2A shows that the mean absolute error per 100 kcal was largest in the “Beverages” category, with a mean error of 0.20 g per 100 kcal. Mean errors in all other categories of interest were negative, indicating an underestimation of added sugar values.

### 3.2. Accuracy of Predicting Added Sugar Using the XGBoost Algorithm

The results of our grid search indicated that an XGboost model trained with a maximum tree depth of 20, a learning rate of 0.005, a minimum child weight of 3, a subsample ratio of columns of 0.9, and a subsample ratio of rows of 0.5 and 2000 trees performed best to predict the added sugar values of foods. Using these hyperparameters, we trained an XGBoost model on our training data and predicted added sugar values in the test dataset.

The evaluation of the resulting model on the test dataset showed that 93.25% of the variance per default potion size was explained (84.32% per 100 kcal). The mean value of estimated added sugar was 4.25 g (SD = 7.79) per default portion size and 3.51 g per 100 kcal (SD = 4.96). The mean absolute error of the estimation was 0.84 g per default portion size (0.81 g per 100 kcal). For category labels in our dataset that overlapped with main sources of added sugar as identified by the USDA, the mean value of estimated added sugar was M = 8.48 g (SD = 13.0) per default portion size and M = 7.55 g (SD = 9.17) per 100 kcal for “Beverages”; M = 10.25 g (SD = 6.04) per default portion size and M = 7.18 g (SD = 3.93) per 100 kcal for “Desserts, cakes, candy, cookies”; M = 7.83 g (SD = 5.93) per default portion size and M = 4.42 g (SD = 2.70) per 100 kcal for ”Breakfast cereal, pancakes and waffles”; and M = 2.83 g (SD = 3.68) per default portion size and M = 3.76 g (SD = 4.70) per 100 kcal for “Dairy”. Mean added sugar in the overall test dataset was 4.26 g (SD = 8.14) per default portion size and 3.55 g per 100 kcal (SD = 5.39). The rank correlation comparing actual to predicted added sugar values yielded significant results (Spearman’s rho for default portion size = 0.87 and per 100 kcal = 0.86, *p* < 0.001). Figure 1B shows that errors in grams per 100 kcal were roughly symmetrically distributed around zero with a slight bias towards overestimating added sugar content.

Figure 2B shows that the mean absolute error per 100 kcal was largest in the “Beverages” category, with a mean absolute error of 2.3 g per 100 kcal. A closer examination of the error shows that added sugar content in beverages was often overestimated, which frequently happens in beverages such as 100% fruit juices, which are high in naturally occurring sugar, while having no added sugar. The mean error in all other categories of interest was negative, indicating an underestimation of added sugar in those categories.

**Figure 3:**
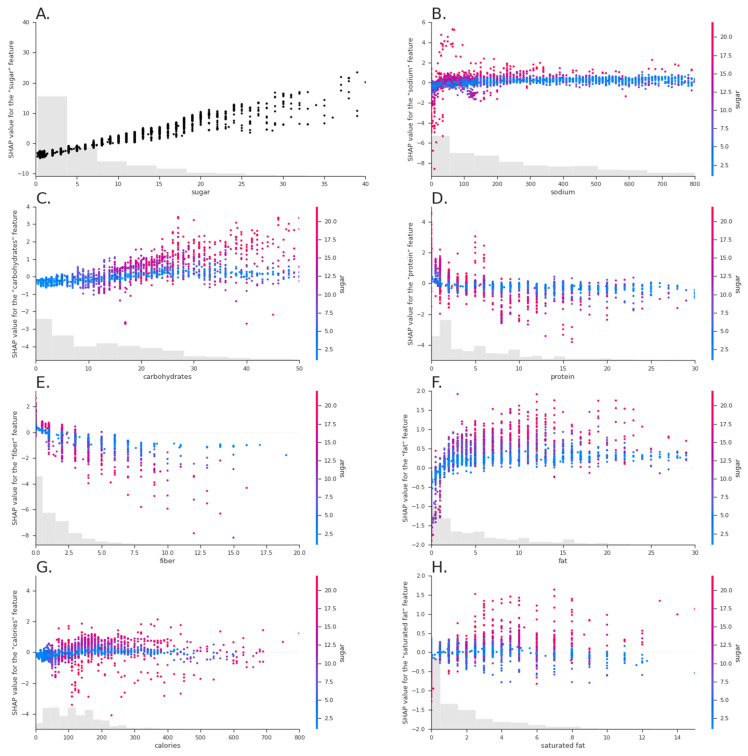
SHAP dependence plots. The value of the feature is displayed on the horizontal axis, while the vertical axis shows how much the value of a feature changed the final model prediction, with each dot representing a specific food. The bars at the bottom of each chart represent histograms showing the distribution of the feature, e.g., for plot A the histogram indicates that most foods in the dataset contain 0 to 5 g of sugar. Blue colors in plots (**B**–**H**) indicate low sugar values, while red colors indicate high sugar values. (**A**) The final model prediction for added sugar depends roughly linearly on sugar. (**C**) For foods with a high value of sugar, the presence of a high amount of carbohydrates indicates higher added sugar values than for foods that are low in sugar. (**D**) Only for high values of sugar does the prediction of added sugar depend on protein. For high values of sugar, lower values of protein indicate higher values of added sugar. (**E**) Low values of fiber indicate higher values of added sugar, especially if the food contains high amounts of sugar.

Investigating the feature importance using SHAP values indicated that, as expected, the value of total sugar was the most important predictor for estimating added sugar content of a food (see Table 1, which shows a summary providing information on how much the value of each feature impacted the prediction). As shown in Figure 3A, the predicted value of added sugar linearly depended on the total sugar content of a food. However, other features also influenced the prediction. As Figure 3D shows, for high values of total sugar, the protein content of a food modulated the prediction. In that case, lower protein content indicated higher added sugar content. Figure 3E shows that lower fiber content also led to higher predictions of added sugar, especially if the total sugar content was high.

## 4. Discussion

We hypothesized that recent advances in computational science, specifically the development of the XGBoost algorithm, can be leveraged to develop an accurate and scalable model to estimate the added sugar content of foods based on their nutrient profile. We further expected the XGBoost approach to surpass previous approaches using the kNN algorithm [15] not only in scalability, but also in accuracy in situations where predictions are to be made on nutrition information imputed by the user directly (and therefore not containing additional information on, e.g., food category, or a list of ingredients). Thus, we trained both a kNN and an XGBoost model to predict the added sugar content of foods based on eight nutrients (sugar, carbohydrates (without fiber and sugar alcohols), fiber, protein, fat, saturated fat, sodium, and calories). We utilized nutrition data for almost seventy-thousand representative foods from the U.S. portion of the WW International Inc. food database, for which added sugar values were available from the nutrition label. Then, 80% of the data were used to optimize and train the models, while 20% of the data were held out to test their accuracy. The models were trained on all foods; however, only foods with non-zero sugar content were included in the test data. The final XGBoost model explained over 93% of the variance in added sugar per default portion size on the test dataset, while the previously reported kNN model explained less than 86% of the variance per default portion size, suggesting that the XGBoost algorithm can provide a more accurate model. The output of the XGBoost model can be helpful to inform consumption choices that enable staying below the 10 percent suggested maximum added sugar intake.

For foods where error was observed in the XGBoost model, there was a slight bias towards overestimating added sugar, which allowed for conservative estimation. Given that consumers tend to underestimate the calorie and nutrient content of foods [19,20], and that average daily added sugar consumption is above the recommended thresholds [4], a slight overestimation of added sugar in the model is preferred relative to underestimation. Among the food categories that are the highest sources of added sugar consumption by Americans, we saw the highest errors for the estimation of added sugar content for beverages. Upon visual inspection, these errors appeared to stem primarily from overestimating added sugar content within fruit juices, which are high in naturally occurring sugar. This is consistent with what was observed within the model, as the value of total sugar is the strongest predictor for estimating the added sugar content of a food. Specifically, the predicted value of added sugar is linear with the total sugar content of the food. Additional predictors for higher added sugar content include low protein content in the food, as well as low fiber content, particularly when controlling for higher total sugar content.

In addition to an error rate in the predictions which was above zero, the presented study has several further limitations. While the general approach presented can be applied to any available dataset, here we were only using the U.S. portion of the WW International Inc. food database to train the model, as reliable information on the actual added sugar content of foods was only available to us in the U.S. when the study was conducted. However, any machine learning model that is trained on a specific dataset can only extract the information that is contained within that specific dataset [17], so it cannot be assumed without further investigation that patterns observed in U.S. packaged foods fully translate to other countries. For example, average added sugar values in packaged foods might be higher or lower in other countries on average. Therefore, if not available on the Nutrition Label, for each country in which a model trained with machine learning is being used for predictions, other sources of truth for added sugar values, such as partial information from the manufacturer or estimates from trained nutritionists, need to be applied to create a new test dataset to evaluate the accuracy of the predictions; if found lacking, the model will have to be retrained with the new data using the same methods as presented in this study.

Furthermore, not all of the eight nutrients used to make added sugar predictions in this study might be available on the label in each country. In these cases, a close evaluation has to be conducted on whether the predictions are still accurate without the missing nutrient as a predictor, and if not, whether an accurate new model can be trained without that predictor or whether it can be imputed using similar methods as presented in this study to increase the accuracy of the added sugar prediction.

The excess consumption of added sugar can contribute toward poor nutrition and a dietary pattern that can increase the risk for obesity, diabetes, and cardiovascular disease in adults and in children as young as seven years old [1,2,3,21]. Currently, the average added sugar consumption is higher than the recommended intake of less than ten percent of daily calories [4]. Given that only a few countries include added sugar in the Nutrition Facts label, a large proportion of the world’s population does not have access to added sugar. As recent data suggest that detailed information about the nutrition content of foods is efficient in guiding consumption behaviors, the results presented herein are important as they can be used to estimate the added sugar of a food from other information provided within a traditional Nutrition Facts label. This demonstrates an opportunity to develop digital solutions, such as a website that estimates the added sugar content of foods, or an app that allows users to scan the traditional nutrition label of food items, and then shares the estimated added sugar content of the item to inform healthy decision making [22]. In the absence of such tools, in countries where added sugar content is not displayed on the nutrition label, consumers are left to determine the healthfulness of a food by estimation alone or by the nutrition claims on the packaging of the food. This is important, as the effectiveness of various sugar label formats can be highly influential on consumer perceptions. For example, a systematic review investigating the impact of nutrition claims related to sugar content on food choices and energy intake suggested that simply writing ‘reduced-sugar’ on a label was enough for health-conscious consumers to perceive a food to be healthier than ‘regular’ foods [23]. Moreover, reporting sugar content in grams along with illustrations and direct statements were determined to be more effective than a standalone Nutrition Facts label [22]. The predictions from the model presented here can be utilized to create accurate and effective information via a mobile app to help inform consumers of added sugar content. Thus, it showcases an opportunity of how data, in combination with predictive modeling, can be leveraged in digital solutions to inform lifestyle choices that can lead to the prevention of chronic noncommunicable diseases. [13]

## 5. Conclusions

We were able to train a gradient boosted tree model to accurately predict the added sugar content of foods, utilizing only information found on a traditional Nutrition Facts label. This will allow for consumer-friendly, digital, and scalable applications to be developed to help inform consumers about added sugars in the foods they wish to consume, even in countries that do not provide added sugar information in the Nutritional Facts label.

## Figures and Tables

**Table 1:** Mean absolute average impact of each feature in the XGBoost algorithm’s output magnitude. The value of sugar is the most important factor in predicting added sugar.

	Mean Absolute SHAP
Sugar	3.20
Sodium	0.61
Fiber	0.43
Protein	0.55
Carbohydrates	0.56
Fat	0.39
Calories	0.30
Saturated Fat	0.15

## Data Availability

The data used in this manuscript are proprietary to WW International, Inc. However, results can be replicated on any dataset that contains nutrition information statements publicly available on food nutrition labels.
